# Dynamic Quantitative Iodine Myocardial Perfusion Imaging with Dual-Layer CT using a Porcine Model

**DOI:** 10.1038/s41598-019-52458-1

**Published:** 2019-11-05

**Authors:** Kai Scherer, Johannes Hammel, Thorsten Sellerer, Korbinian Mechlem, Bernhard Renger, Andrea Bähr, Christian Kupatt, Rabea Hinkel, Julia Herzen, Franz Pfeiffer, Ernst Rummeny, Daniela Pfeiffer

**Affiliations:** 1 Department of Diagnostic and Interventional Radiology, School of Medicine & Klinikum rechts der Isar, Technical University of Munich, 81675 München, Germany; 2 Chair of Biomedical Physics, Department of Physics and Munich School of BioEngineering, Technical University of Munich, 85748 Garching, Germany; 3 Klinik und Poliklinik Innere Medizin I, Klinikum rechts der Isar, Technical University of Munich, 81675 München, Germany; 4 DZHK (German Centre for Cardiovascular Research) - partner site Munich Heart Alliance, 80802 Munich, Germany; 5 Department of Laboratory Animal Science, Deutsches Primatenzentrum GmbH, Leibniz-Institut für Primatenforschung, 37077 Göttingen, Germany; 6 Institute for Advanced Study, Technical University of Munich, 85748 Garching, Germany

**Keywords:** X-rays, Cardiology, Cardiovascular diseases

## Abstract

Ischemic heart disease is the globally leading cause of death. When using coronary CT angiography, the functional hemodynamics within the myocardium remain uncertain. In this study myocardial CT perfusion imaging using iodine contrast agent demonstrated to strongly improve the assessment of myocardial disorders. However, a retrieval of such dynamics using Hounsfield units from conventional CT poses concerns with respect to beam-hardening effects and low contrast-to-noise ratio (CNR). Dual-energy CT offers novel approaches to overcome aforementioned limitations. Quantitative peak enhancement, perfusion, time to peak and iodine volume measurements inside the myocardium were determined resulting in 0.92 mg/ml, 0.085 mg/ml/s 17.12 s and 29.89 mg/ml*s, respectively. We report on the first extensive quantitative and iodine-based analysis of myocardial dynamics in a healthy porcine model using a dual-layer spectral CT. We further elucidate on the potential of reducing the radiation dose from 135 to 18 mGy and the contrast agent volume from 60 to 30 mL by presenting a two-shot acquisition approach and measuring iodine concentrations in the myocardium *in-vivo* down to 1 mg/ml, respectively. We believe that dynamic quantitative iodine perfusion imaging may be a highly sensitive tool for the precise functional assessment and monitoring of early myocardial ischemia.

## Introduction

In 2015 approximately 15.9 million myocardial infarctions occurred worldwide^1^. Thereby ischemic heart disease is the most common cause of death (7.249 million deaths worldwide in 2008) accounting for 12.7% of the total global mortality^2^. Further myocardial ischemia is responsible for angina, unstable angina as well as cardiac arrhythmias.

While coronary computed tomography angiography (CTA) shows high sensitivity towards the anatomical detection of destructive coronary artery disease - which is the most frequent reason for myocardial infarction - the diagnosis of myocardial ischemia itself is rather unspecific, contingent on the lower positive predictive value of obstructive lesions detected by the latter^3^. Consequently, the number of invasive coronary angiography and revascularization procedures is considered higher than necessary^4,5^. To overcome these shortcomings and assess myocardial hemodynamics in a functional, i.e. not purely anatomic manner, dynamic imaging techniques such as cardiac perfusion CT and cardiac magnetic resonance imaging (CMR) perfusion have evolved. While perfusion CMR is a well-established technique for the detection of myocardial ischemia without radiation exposure for patients, CT offers several advantages compared to MR. First, a CMR examination of the myocardium takes 45 to 60 minutes, whereas CT of the heart including calcium scoring, CCTA and perfusion analysis can be performed within 10 minutes. Especially in an emergency setting this is of special importance. Short scan time in CT also helps to avoid movement artifacts. In general, a main advantage of CT is its high resolution. Considering the relatively small thickness of the myocardium, this has a high potential to improve the detection of even small regions of subendocardial ischemia. Multiple studies showed evidence that myocardial perfusion imaging can significantly increase the accuracy of assessing flow limiting coronary artery diseases or areas of prior infarction^6–8^. Especially in the case of moderate coronary artery disease, where an anatomical evaluation of the stenosis is ambiguous, perfusion imaging is considered helpful for diagnosis.

Among CT myocardial perfusion (CTMP) imaging two approaches have been developed. With the static technique a single scan is performed when the enrichment of iodine within the myocardium is considered maximal, so that an optimal contrast between healthy, under-perfused and infarcted myocardial tissue is achieved. To ensure correct timing, the (test) bolus has to be tracked and the enrichment within the aorta determined^9^. The major advantage of static perfusion imaging is that myocardial blood-flow can be rapidly assessed, and that the underlying radiation dose is comparatively low. Using rest/stress protocols, especially in combination with CTA, a good assessment of suspected coronary artery disease has been reported^10,11^. However, static CTMP only provides a (semi-)qualitative analysis, while also being highly susceptible to temporal perfusion artifacts and thereby heavily reliant on the correct timing. In comparison, dynamic CTMP is performed by constantly acquiring scans within a 20–40 seconds timeframe and hence imaging the complete first pass of contrast agent through the heart^12^. While this technique is rigid and allows for a quantitative analysis of hemodynamics, the underlying Hounsfield-based assessment is susceptible to beam-hardening effects induced by the iodine contrast agent^13^. Further physiological models must be considered which relate Hounsfield units to the actual blood or iodine flow^14^. Finally, the continuous acquisition mode poses concerns with respect to an increased radiation dose.

Here the introduction of dual-energy CT (DECT) in clinical routine offers potential to overcome the aforementioned limitations. Different technological approaches have been established for acquiring dual-energy data. Mainly three technical implementations are used in clinical CT scanners: Dual-source CT (DSCT), rapid kVp switching CT (KVSCT) and Dual-layer CT (DLCT). A DSCT system consists of two X-ray tubes and two associated detectors, whereas the KVSCT machines use one detector and a source with two alternating kVp settings during the scan. To acquire spectral information in DSCT and KVSCT systems a scan must be designed accordingly before its start. As the photon energy separation takes place in the detector, in DLCT conventional and spectral data are obtained simultaneously without the necessity of a spectral scan pre-setting. Spectral data provides strongly increased contrast and sensitivity (via the virtual monoenergetic image (MonoE) and iodine density image channels) towards soft-tissue and iodine-based imaging, thereby opening up new perspectives for a reduction of radiation dose and volume of injected contrast agent. In 2012 So. *et al*. showed that by using rapid kVp switching DECT a higher reproducibility of dynamic myocardial perfusion measurements in comparison to a Hounsfield units based evaluation can be assured^15^. Further Sánchez-Gracián *et al*. demonstrated, that iodine quantification in myocardial perfusion stress DECT is beneficial for the differentiation of healthy and ischemic or necrotic myocardium^16^. Finally, Fahmi *et al*. reported on quantitative myocardial perfusion images and thereby demonstrated a superior assessment of myocardial infarcts in comparison to a conventional measurement^17^. Sellerer *et al*. showed an improved root mean square deviation (RMSD) of observed iodine concentrations (with respect to true values) for different measurement configurations using a modern clinical DLCT system^18^. This is of particular importance, as the quantification accuracy of iodine densities in regions of low contrast agent accumulation like in the myocardium plays a crucial role in the generation of our results.

In this study, we report on the very first extensive quantitative and iodine-based analysis of myocardial dynamics in a healthy porcine model in rest, using a dual-layer spectral CT. We include the presentation of iodine perfusion, time to peak, peak enhancement and volume maps. In a first step, we show in congruence with previous studies, that low-energy virtual MonoE-keV images and iodine density maps are superior in comparison to conventional images in the depiction of temporal opacification of the myocardium with respect to relative signal enhancement and contrast-to-noise ratio. Afterwards the temporal uptake of iodine is modelled using a gamma-variate function and corresponding dynamics deduced from the model in a pixel-wise manner. Further, we elucidate on the possibility and performance of quantitatively assessing the myocardial peak enhancement map using a semi-static two-shot approach. Based on the accuracy and consistency of determining low iodine densities with modern dual-energy systems, we suggest that dynamic quantitative iodine perfusion imaging may be a highly sensitive tool for the precise functional assessment and monitoring of early myocardial ischemia, if a valid database for stress and/or rest can be established^18^.

## Results

**Figure 1:**
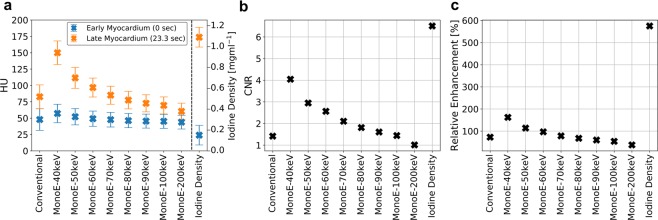
(**a**) Conventional and mono-energetic Hounsfield units and iodine density of the early (blue points) and late myocardium (orange). The early stage is measured at second zero without iodine enhancement, the late myocardial stage is depicted at second 23.3 with maximal contrast agent enrichment. Error bars correspond to the standard deviation found within a ROI of 50 mm^2^ of the respective image channels. (**b**) Temporal contrast-to-noise and (**c**) relative signal enhancement of the myocardium during contrast uptake derived from (**a**), demonstrating a superior depiction of the myocardial opacification within the iodine density imaging channel.

A healthy pig (31.75 cm in mean diameter, 78 kg) was scanned using a state of the art dual-layer spectral CT system from Philips with a detector coverage of 4 cm and a peak voltage of 120 kVp. An overall volume of iodine contrast agent of 40 ml (Ultravist 300, Bayer, Bayer AG, Leverkusen, Germany) was injected into the ear vein, which corresponds to a concentration of 154 mg/kg-bodyweight. Within this animal study, scans were immediately acquired after injection to track the bolus and enable hemodynamics analysis throughout the whole thorax. Electrocardiographically (ECG)-triggered dynamic axial scans were adjusted to the cardiac cycle with an increment of zero, with the scanning area covering the mid part of the left ventricular myocardium. A total of 36 consecutive scans were acquired. Finally, no (commercial) automatic motion-correction algorithm for the heart was available as the entire heart volume was imaged only fragmentary. Therefore, data points with extensive motion in heart or other thorax structures were manually abandoned, resulting in a non-equidistant sampling of the curve with 15 scan points. Figure 1a shows the corresponding Hounsfield units for conventional and mono-energetic reconstruction as well as the iodine density of the porcine myocardium at the initial stage and at high contrast agent enrichment, i.e. 23.3 seconds after the contrast agent injection. In congruence with previous dual-energy studies on other indications, the temporal relative signal increase during iodine uptake with respect to image noise is highest in case of the low mono-energetic channels as well as the iodine density map itself^19,20^. This is also well reflected in the temporal contrast-to-noise ratio (Fig. 1b) and relative signal enhancement (Fig. 1c), where the virtual MonoE-40 keV and iodine density images outperform the conventional HU image by far.

**Figure 2:**
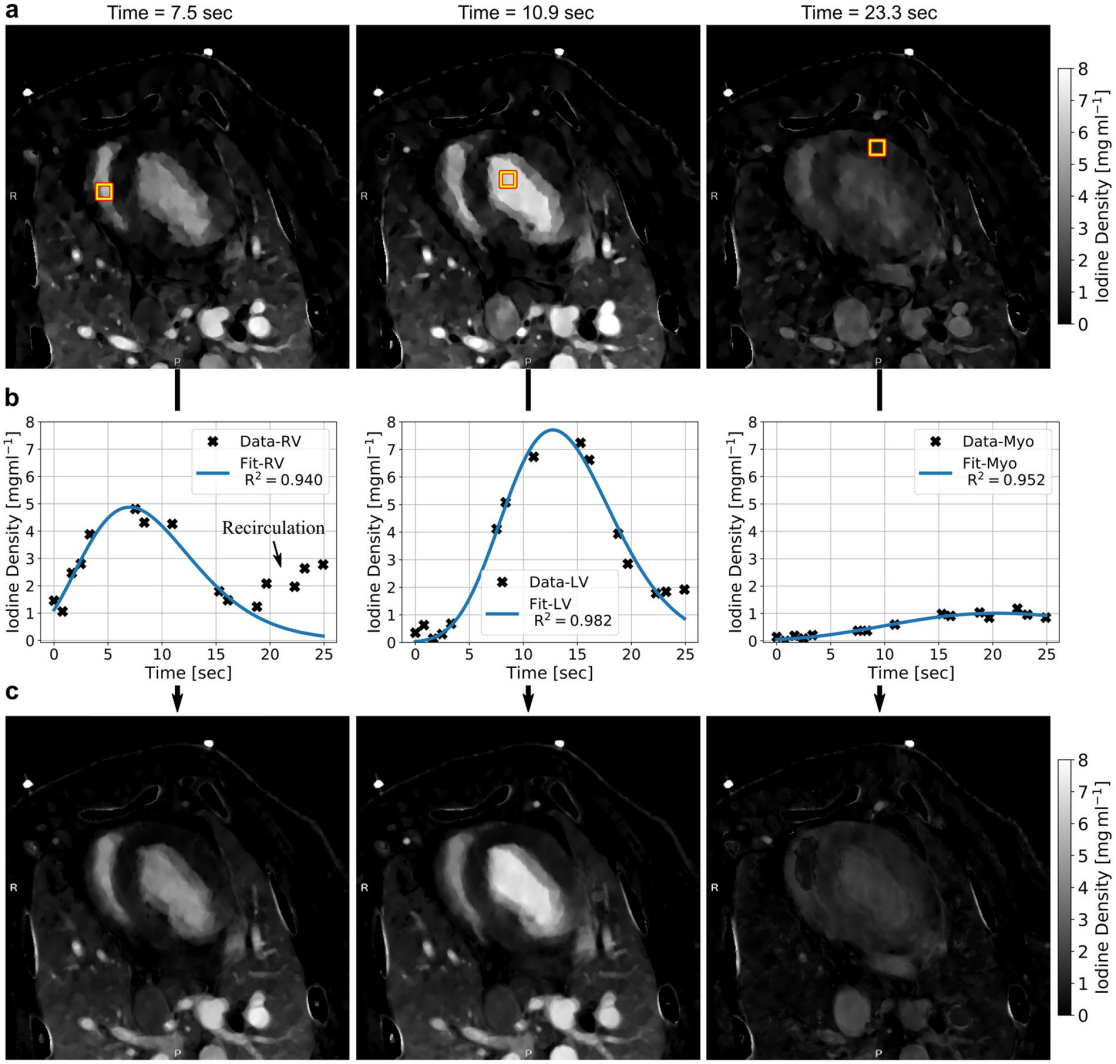
(**a**) Measured transversal quantitative iodine density slices of the porcine thorax showing a subsequent opacification within the right (RV) and left heart ventricle (LV) as well as the myocardium (Myo). (**b**) Iodine density values vs. scan time as obtained from the dynamic dual-energy perfusion CT for an exemplary pixel for various region of interest as indicated by yellow boxes in (**a**), which were selected by an experienced radiologist. A high accordance of data and fit was obtained using a gamma variate fit model. Note that only the first pass of contrast agent was considered, and a recirculation of blood was correspondingly neglected for the fit. (**c**) Transversal quantitative iodine density slices of the porcine thorax obtained from the model being in good agreement with the measured data as shown in (**a**). Note that distinctive discrepancies for late scan times arise from the neglection of recirculation.

Since the temporal opacification of the myocardium is quantitatively and most sensitively depicted (as an iodine density offset arising from soft tissue is nearly absent) within the iodine density map, this channel was used for further iodine-dynamics analysis. Figure 2a displays a transversal porcine thorax slice for different scan times after the initial injection of contrast agent. Notice a subsequent bolus pass/enrichment of iodine within the right and left heart ventricle as well as myocardium for the exemplary scan times of 7.5 seconds, 10.9 seconds and 23.3 seconds, respectively. In a first step, imaging data of 15 scan points was temporally modelled in a pixel-wise manner using gamma variate fit functions, as proposed in literature^21,22^. Using a two-step fitting routine, only the first pass of contrast agent was considered so that artifacts arising from recirculation were excluded. Figure 2b displays data-points and the corresponding fit for an exemplary pixel in various regions of interest (ROI) showing a high degree of correlation of model and measurements (as quantified by the coefficient of determination R^2^ being close to 1), even in case of a non-equidistant temporal sampling. The corresponding transversal thorax slices obtained from the model (Fig. 2c) are of smoother appearance than the original data, contingent on the fact that outliners and noise are damped via the fitting routine. This however is an essential premise for the meaningful retrieval of perfusion and peak enhancement map. Please note distinctive discrepancies between model and measured data for late scan times arising from a correction of recirculation, as indicated by low iodine density areas, among others, in the right ventricle.

**Figure 3:**
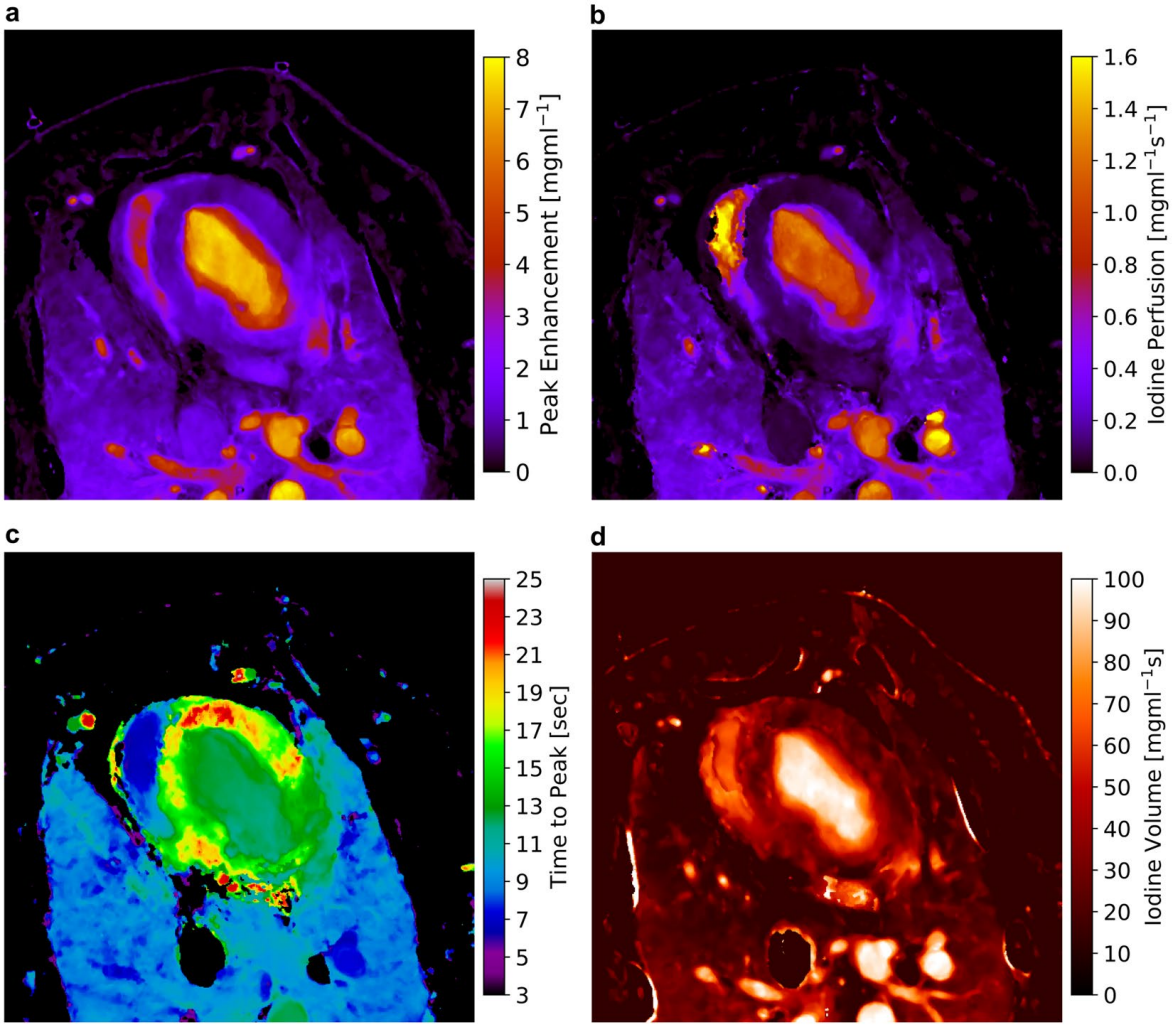
Absolute and quantitative iodine-based analysis of myocardial dynamics: (**a**) peak enhancement, (**b**) perfusion, (**c**) time to peak and (**d**) volume maps of the porcine thorax, derived from the gamma variate fit model using the slope method. The peak enhancement indicates the maximal increase in iodine density, the perfusion relates to the highest temporally gradient in influx of iodine, the time to peak indicates the point in time when maximal peak enhancement is reached, and the iodine volume is related to uptake/storing/flush out behaviour within the respective structures. Note that areas, where the gamma variate fit failed (for instance in the cava inferior) or areas where the iodine density remained below 0.55 mg/ml during the scan are color-coded black.

In a next step the fitted data was used to derive the absolute, quantitative iodine peak enhancement, perfusion, time to peak and volume maps as depicted in Fig. 3, using the maximal slope method^23^. A peak enhancement value (Fig. 3a), which is given as the maximal absolute increase in iodine density within the measured time frame, of 0.92 mg/ml (11,86% of the value found in the descending aorta) was determined within the myocardium. The corresponding perfusion value (Fig. 3b), i.e. the maximal slope of the temporal iodine density curve, was determined to 0.085 mg/ml/s. This value amounts to only 6,7% of the value found within the descending aorta, contingent on a limited influx of contrast agent through the coronary arteries in combination with a slow uptake/diffusion within the dense muscular tissue. Interestingly similar values were found in the ventral region of the lungs. As expected for a healthy animal, the time to peak map (Fig. 3c) indicates a simultaneous and consistent perfusion throughout the entire myocardium, approximately 4–5 seconds after the bolus passes through the descending aorta. Only a small ventral region with a delayed maximal opacification was found. However as depicted in the very right line plot in Fig. 2b the iodine density itself only yields minimal changes within the timeframe of 16–24 seconds. Finally, the overall volume of iodine perfusing the porcine thorax was calculated by integrating the temporal iodine density curves. As the myocardial enrichment remains high for late scan times and a flush out of iodine from the muscle is relatively slow in comparison to vascular and ventricular heart structures, the fitted curves were integrated within a timeframe of 50 seconds (so that the iodine density drops below a threshold value of 0.25 mg). Here a value of 29.89 mg/ml*s was calculated within the myocardium which accounts to 29,43% of the volume dwelling in the aorta. An overview of all perfusion parameters for various exemplary locations in the thorax is given in Table 1.

**Table 1:** Quantitative iodine-based peak enhancement, perfusion, time to peak and iodine volume of the myocardium in comparison to heart, lung and arteries.

	Peak Enhancement [mgml^−1^]	Iodine Perfusion [mgml^−1^s^−1^]	Time to Peak [sec]	Iodine Volume [mgml^−1^s]
Myocardium	0.92	0.085	17.12	29.89
Left Ventricle	7.52	1.269	12.39	96.77
Right Ventricle	3.77	1.561	6.72	62.73
Descending aorta	7.76	1.232	12.26	98.19
Ventral Lung	1.01	0.19	8.66	17.26
Pulmonary artery	6.92	1.25	9.83	95.93

The simultaneous analysis of all perfusion parameters allows for a specific diagnosis of myocardial disorders: a reduced peak enhancement with a normal time-to-peak value indicates scarred/fibrotic myocardium, limiting the absolute capacity of the myocardium, while in presence of a delayed peak value an ischemic disorder is considered^24^. Further, the perfusion pattern, which is related to the temporal change of blood flow in both volume and velocity, is thereby a good indicator for a blocked or limited supply of blood through the coronary arteries. Therefore, quantitative CT perfusion of the myocardium assessed by absolute iodine concentration measurements over time has the potential to significantly increase the performance of CT for detection of myocardial ischemia in patients with ischemic heart disease. The blood volume (or in this case the iodine volume map), gives additional information on the overall blood intake capacity, its storing as well as the flush out behaviour.

**Figure 4:**
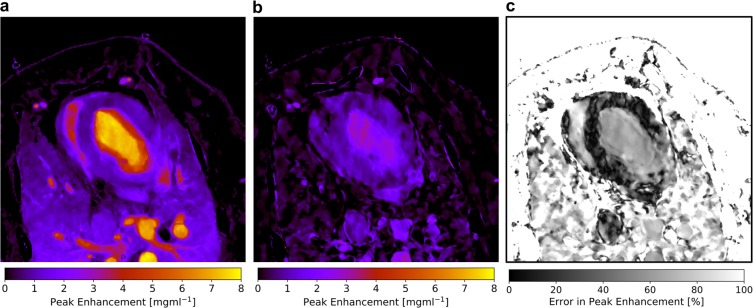
(**a**) Quantitative iodine-based peak enhancement of the porcine thorax as derived from the dynamics analysis based on 15 scans. (**b**) Quantitative iodine-based density peak enhancement of the porcine thorax as derived from a semi-static two-shot approach during myocardial contrast saturation. Notice that arterial and ventricular structures are strongly underestimated in comparison to (**a**). (**c**) Relative error in the determination of the peak enhancement of (**b**) in comparison to the ground truth (**a**). Within the myocardium the deviation is quite homogeneously patterned and accounts to values in the range of 5–15%.

A major drawback of a precise flow dynamics analysis is the necessity of a sufficient temporal sampling of the iodine density curve. This implies acquisitions before and during iodine contrast enrichment until myocardial saturation is reached, which poses concerns with respect to radiation dose. For an initial assessment of the myocardial blood flow (for instance in case of a screening modality or when myocardial ischemia is uncertain), we propose to derive the quantitative, iodine peak enhancement map from a semi-static two shot approach in stress and/or rest without the need of modelling/fitting the data. For this, two scans are obtained in the myocardial saturation phase (in this case at 23.3 and 24.9 seconds). To obtain a homogeneous pattern within the myocardium, the corresponding two iodine densities are averaged and smoothed by a minor Gaussian-blur (to mimic the fitting routine). Finally, to directly calculate the peak enhancement, an offset value from literature or a scout scan/control measurement is subtracted. Note that in a conventional HU-based assessment an arterial curve must be obtained in order to normalize data, which however is obsolete in case of quantitative, absolute iodine density values. Figure 4a,b compare the ground truth peak enhancement map of the fully sampled scan with the one obtained by the two-shot acquisition method, respectively. Obviously, the peak enhancement values within the ventricles and arteries is underestimated by far. However, in case of the saturated myocardium the two-shot approach yields very similar values to the model. The relative error within the peak enhancement (Fig. 4c) accounts to values in the range of 5–15% and is considerably homogeneous within the overall myocardium. Note that the remaining dynamics parameters as derived by the maximum slope method remain unassessed as here the precise onset as well as saturation of iodine throughout the myocardium must be tracked in time.

## Discussion

Within this study, we showed that by using a state-of-the-art dual-energy perfusion CT imaging technique, it is possible to deduce absolute iodine-based flow dynamics of the heart, arteries and myocardium. This allows for an assessment of functional properties of the latter in a quantitative manner. Thereby, major advantages arise in comparison to conventional HU-based diagnosis: first of all, the increase in image contrast during perfusion is most pronounced in the iodine density map, helping with a better visual assessment of the myocardium and potential ischemic or infarcted regions. This allows for a more rigid fit of the imaging data as well as the potential to reduce the amount of contrast agent and radiation dose. Secondly, the image values examined by the radiologist are not affected by beam hardening artifacts and hence are independent from factors such as patient size and variations between different CT platforms, facilitating a value-based assessment. Finally, the obtained image data is quantitative, which renders an acquisition and consequent normalization with an arterial curve unnecessary, provided that the concentration of contrast agent with respect to patient characteristics is reliably controlled. Given the establishment of an extensive clinical database comparing iodine-based dynamical indicators for various myocardial dysfunctions, in both stress and rest, we believe that DECT may allow for a fast, rigid, highly sensitive and specific assessment of heart diseases.

However, an essential requirement for the establishment of such a database and using data without normalization is that the quantitative iodine densities present in the human myocardium can be measured precisely and in a reproducible manner. In the here presented animal study, a volume of 40 ml of Ultravist 300 was injected and an offset adjusted iodine density of 0.92 mg/ml found within the myocardium. Note that the corresponding iodine *in-vivo* concentration of 154 mg/kg-bodyweight, is well below volumes typically applied in clinical routine. In a very recent study comparing various dual-energy scanners Sellerer *et al*. demonstrated, by using an abdominal phantom and mimicking various patient sizes, that even low iodine concentrations below 1 mg/ml (dependent on underlying dual-energy technique and patient size) can be reliably measured with a relative error around 10% only^18^. Older studies reported on similar values, i.e. a constant absolute error of 0.1 mg/ml in case of iodine concentrations below 1 mg/ml^25^. Given a relative increase of iodine density within the healthy myocardium of 575% (0.16 mg/ml to 1.08 mg/ml) during contrast uptake within our study, the accuracy of modern systems can be considered as sufficiently high.

We further demonstrated that a semi-static two-shot approach yields a deviation of 5–15% in the iodine peak enhancement value in comparison to one derived from the full scan. Ensuing from previous studies showing that ischemic and infarcted regions of the myocardium exhibit a by 23% and 47% decreased mean iodine density, we believe that the quantitative semi-static approach may not be highly specific, however may be a good first indicator for myocardial malfunction (e.g. in case of a reduced peak enhancement or delayed time to peak value) in an initial assessment^16^. Further, previous studies on quantitative iodine perfusion implied the usage of 60 mL of Ultravist 370 (approximate iodine concentration of 317 mg/kg-bodyweight) resulting in an iodine density of 2.56 mg/ml within the healthy myocardium^16^. As we found a value of approximately 1 mg/ml in the myocardium (iodine concentration of 154 mg/kg-bodyweight) which can be reliably measured with modern DECT systems and allows for a meaningful modelling of the data, we believe that a reduction of applied iodine volume/concentration by a factor of 2 is possible.

Finally, in this animal study 15 scan points were used for the analysis, in order to model early enrichment of arteries and ventricles, accounting to a computed tomography (CT) dose index (CTDIvol) of 15 × 9 mGy equalling 135 mGy. In a clinical setting when only the myocardium is of interest early scan times after the injection can be neglected and an equidistant sampling of myocardial perfusion slope and saturation with 8–12 points is generally sufficient. Correspondingly a CTDIvol of 72–108 mGy can be expected, being well below a critically considered value of 250 mGy.

The purpose of this experiment is considered a proof of principle investigation. As the DECT technique offers several advantages regarding quantitative image results using iodine contrast agent, the main point of our manuscript focuses on a initial investigation of quantitative perfusion measurements. The added value of this study is to show the advantage of quantitative iodine density maps in comparison to conventional CT images. In terms of the improved contrast-to-noise ratio and the relative signal enhancement using contrast agent density maps in DECT perfusion imaging, our results are relevant to several clinical perfusion use cases with animals or even humans. Indeed, the improvement of clinical diagnosis should be considered in further statistically meaningful studies with the benefit of various patient groups and myocardial disorders in rest and stress condition. This will permit the establishment of a valid database of quantitative iodine-based hemodynamical parameters. Hereby it will be of essential importance to clarify which of the quantitative hemodynamical indicators are of major diagnostic importance. Further, within this study a dual-layer IQon Scanner from Philips was used, providing a detector coverage of 4 cm only. A spatially limited examination of the heart (especially the myocardium) however bears the risk of overlooking locally confined flow defects. Under these circumstances, vendors are obligated to increase detector coverage in order to screen the entire myocardium at once.

## Materials and Methods

### Ethics statement

Animal care and all experimental procedures were performed in strict accordance to the German and National Institutes of Health animal legislation guidelines and were approved by the Bavarian Animal Care and Use Committee (AZ 55.2.-1-54-2532-62-13).

### Acquisition of CT myocardial perfusion

CT images of the healthy pig (31.75 cm mean diameter, 76 kg) were acquired with a 64-slice single source dual-layer spectral CT IQon scanner with a detector coverage of 4 cm and a rotation time of 0.27 seconds (Philips Healthcare, The Netherlands). The spatial resolution is 2.47 pixels per mm resulting in a pixel size of 0.40 × 0.40 *mm*^2^. The ECG triggered temporal resolution aggregates to 1.08 scans per second or a full acquisition of the specified range every 0,92 second. Firmware version on the scanner was 4.1.0.0. The perfusion scan was conducted in stationary mode with 120 kVp and a mean tube current of 100 mAs. Scans were reconstructed with a Philips B-kernel and a slice thickness of 3 mm. Within 26 seconds 36 scans were recorded, so that the full first pass of contrast bolus was imaged. Hereby the scans were electrocardiographically (ECG)-triggered and adjusted to the cardiac cycle with an increment of zero. Each scan was conducted with a CTDIvol of 9 mGy, resulting in a CTDIvol of 135 mGy and 18 mGy for the extensive analysis of myocardial dynamics (15 scan points) and the two-shot approach, respectively. The perfusion scan was conducted using 40 ml of contrast agent (Ultravist 300, Bayer, Bayer AG, Leverkusen, Germany, iodine content 300 mg/ml) at a flow rate of 4 ml/s. The contrast agent was injected into the ear vein via an 18-gauge catheter using a dual syringe injection system (Stellant, MEDRAD, Inc., Indianola, PA, USA).

### Contrast-to-Noise and relative signal enhancement

Conventional, virtual Mono-keV and Iodine density reconstructions alongside with their standard deviations (within respective ROIs of 50 mm^2^) were directly obtained from the Philips IntelliSpace Portal. The relative increase in signal (RIS, Fig. 1c) and temporal contrast-to-noise ratio (CNR, Fig. 1b) during contrast uptake, were calculated as

1 $${\rm{RIS}}=\frac{{S}_{t=23.3s}-{S}_{t=0s}}{{S}_{t=0s}}$$

and

2 $${\rm{CNR}}=\frac{{S}_{t=23.3s}-{S}_{t=0s}}{\sqrt{{\sigma }_{t=23.3s}^{2}+{\sigma }_{t=0s}^{2}}},$$

respectively. Here ${S}_{t=23.3s}$ and ${S}_{t=0s}$ correspond to the different signals within each of the image channels at maximal/saturated and minimal iodine concentration within the myocardium, respectively.

### Retrieval of myocardial dynamics

A certain slice within the thorax was determined for further processing and 15 data points in time, with minor structural movement, were selected from the iodine density images. In order to model the first pass of contrast bolus through the heart, arteries and myocardium, gamma variate functions were used, as proposed in literature for both magnetic resonance imaging (MRI) and CT^21,26^:

3 $$I(t)=A{(t-{t}_{0})}^{\alpha }{{\rm{e}}{\rm{x}}{\rm{p}}}^{\frac{-(t-{t}_{0})}{\beta }}$$

whereas I(t) is the time dependent iodine density and *A*, *t*_0_, *α*, *β* are fit parameters. Thereby the data was fitted in a two-step sequence: in a first fit all 15 data points were used and a preliminary peak time determined. In a second fit data exceeding the initial peak by more than 12 seconds, i.e. accounting to the second pass of contrast agent, was correspondingly neglected. Afterwards quantitative iodine perfusion, peak enhancement, time to peak and iodine volume (in imitation of the conventional blood volume) were calculated based on the maximal slope method^23^:

4 $${\rm{Perfusion}}=\frac{d}{dt}{[I(t)]}_{max}$$

5 $${\rm{Peak}}\,{\rm{Enhancement}}={I}_{max}-{I}_{min}$$

6 $${\rm{Time}}\,{\rm{to}}\,{\rm{Peak}}=t({I}_{max})-t({I}_{min})$$

7 $${\rm{Volume}}={\int }_{0}^{50s}I(t)dt.$$

Finally, aforementioned maps were filtered with a median-filter with a 3 × 3-kernel to reduce noise arising from the pixel-wise assessment. Note that data was not normalized or scaled with respect to arterial input functions, hemocrit-scale, cerebral blood volume (CBV) factor or other physiological models for multiple reasons: first of all, within this animal study corresponding values and models are not available. Secondly, we wanted to provide absolute and quantitative, i.e. unscaled, values to the readers, which are independent from assumptions made in underlying physiological models. Correspondingly the iodine volume map presented here does not represent iodine volumes in the sense of millilitres, but rather gives quantitative measure on the area under the curve of the temporal iodine density in the unit of mg/ml*s. The latter is thereby related to the tissues overall blood intake capacity, its storing as well as the flush out behaviour.

## Data Availability

The data generated in the current study is available from the corresponding author on reasonable request.
